# Multitasking: Making the Most out of the Retroviral Envelope

**DOI:** 10.3390/v2081571

**Published:** 2010-08-02

**Authors:** Mariana Varela, Massimo Palmarini

**Affiliations:** 1 Department of Veterinary Medicine, University of Cambridge, Madingley Road, CB3 0ES,Cambridge, England, UK; E-Mail: mv307@cam.ac.uk; 2 Medical Research Council - University of Glasgow Centre for Virus Research, Institute for Infection, Immunity and Inflammation, College of Medical, Veterinary and Life Sciences, University of Glasgow, Scotland, UK

**Keywords:** retroviruses, immune suppression, envelope

## Abstract

Evasion of the host’s immune system is a required step for the establishment of viral infection. In this article, we discuss the recent findings of Heidmann and colleagues demonstrating that some retroviruses possess an immune suppressive (IS) domain “encrypted” within their envelope glycoprotein that is required to establish a successful infection in immunocompetent hosts [1].

The relationship between virus and host is dynamic, with both parties aiming for survival and engaging on an arms race. Viruses have developed a variety of mechanisms to evade the host’s immune system. Some viruses, like herpesviruses and poxviruses, possess large genomes and encode multiple proteins specifically dedicated to silence the host’s immune system [2]. Other viruses make use of fast replication rates and produce variants that escape adaptive immune responses (e.g. hepatitis C virus). Retroviruses use a variety of strategies to counteract the host’s immune system. First of all, retroviruses establish persistent infections by integrating their genome permanently into the host’s genomic DNA. This step assures their survival for as long as the cell lives. Second, some retroviruses like HIV are able to escape the host's adaptive immune responses thanks to their genetic diversity induced by the error-prone reverse transcriptase and a high replication rate. HIV also encodes some accessory proteins (e.g. Nef, Vif, Vpu and Vpr) that are involved in the modulation of the intrinsic, innate and adaptive immune system of the host [3,4]. Interestingly, also the retroviral envelope (Env) protein can play a major role in modulating the immune response of the host. The main function of Env is to mediate entry into the cell by binding to specific cellular receptor(s) and fusing with the cell membrane. However, the Env of some retroviruses have evolved also other functions. For example, the Env of Jaagsiekte sheep retrovirus (JSRV) can function as a dominant oncoprotein and induces cell transformation both *in vitro* and *in vivo* [5,6].

Schlecht-Louf and colleagues have recently described an elegant study addressing the biological role of an immunosuppressive factor encrypted in the envelope glycoprotein (Env) of certain retroviruses [1,7–9] (Figure 1). The immune-modulator function of some retroviruses was first noted in the early sixties, when it was described that both humoral and cell mediated immunity was impaired when mice were infected with Gross passage A leukemia virus [10]. Subsequent studies, both *in vivo* and *in vitro*, allowed the identification of a highly conserved region within the transmembrane domain of the Env protein of murine retroviruses [11]. The use of a synthetic peptide (referred to as CKS-17) designed on the basis of this conserved region, helped to assign many immune-modulator functions to the immune suppressive (IS) domain. CKS-17 was shown to inhibit proliferation of the IL-2 dependent cell line CTLL-2 and alloantigen-stimulated proliferation of murine and human lymphocytes [12]. In addition, CKS-17 was also shown to suppress the respiratory burst of human monocytes and to modulate natural killer cell activity [13,14]. Thierry Heidmann’s group later developed an assay that allowed to demonstrate that the Env of different retroviruses (such as Moloney murine leukemia virus, Mason-Pfizer monkey virus and HERV-H) containing the IS domain are immune suppressive *in vivo* [7,8,15]. The rationale of the assay is that allogeneic tumor cells are usually rejected by the immune system when engrafted into immune competent mice. However, successful engrafting occurs when these cells stably express immune suppressive molecules such as a retroviral Env proteins containing the IS domain.

Interestingly, the IS domain is also present in the Env proteins of some endogenous retroviruses. Endogenous retroviruses (ERVs) have colonized the genomes of all animal species where they account for a substantial portion of their genome (e.g. 8% of the human genome) [16]. The open reading frames of certain ERVs have been preserved for millions of years suggesting that they have co-evolved with their hosts to provide beneficial functions. In the last years, ERVs have been shown to be essential for the reproductive biology of several species including mice, sheep and humans. The sheep genome harbors at least 27 copies of ERVs, referred to as enJSRVs, which are highly related to the exogenous and pathogenic JSRV [17, 18]. enJSRVs are abundantly expressed in the epithelium of the female reproductive tract and in the trophectoderm cells. Using morpholino antisense oligonucleotides *in utero*, it has been shown that expression of enJSRVs Env is absolutely required for conceptus development and placental morphogenesis *in vivo* [19,20]. Both humans and mice possess ERVs expressing highly fusogenic Env proteins in the placenta [21,22]. The use of knockout mice for one of these proteins (S*YNCYTIN-A*) showed that also in this species ERVs are essential for placentation [23]. Expression of the Env glycoproteins of similar ERVs (*SYNCYTIN-1, SYNCYTIN-2* and ERV-3) has been shown also in the human placenta and directly linked to the differentiation and fusion of cytotrophoblasts *in vitro* [24–27].

Given the functional links between retroviral Env proteins and immunosuppression, it was also hypothesized that those endogenous retroviruses possessing an Env with an IS domain could play a role in maternal-fetal tolerance in the placenta. Heidmann’s group experimentally addressed this issue by using the *in vivo* tumor-rejection assay. These authors showed that SYNCYTIN-2 and ERV-3 are immune suppressive while SYNCYTIN-1 is not. In addition, the authors identified key residues in Env that are critical for the IS function but that do not affect the fusogenic activity of these proteins [9].

Despite all the evidence accumulated over the years, the true biological role played by the IS domain remained somewhat unclear, mainly because the experimental systems available did not allow to test the activity of this domain in the context of a fully replicating virus. However, in an elegant study published recently in the *Proceedings of the National Academy of Sciences*, the Heidmann’s group has established the role of the IS domain in viral replication *in vivo* [1]. The authors used Friend murine leukemia virus (F-MLV) and a viral mutant with two key residues within the IS domain replaced by the corresponding residues from *SYNCYTIN-1* (that has no IS activity). The resulting double mutant (DM) displayed the same infectivity of wild type F-MLV in pseudotype assays and it was immune-suppression negative when tested in the *in vivo* tumor-rejection assay. Infection of Swiss mice with wild type F-MLV or the DM virus demonstrated that those two residues in the IS domain are critical for viral replication since no viremia was detected in animals infected with the DM envelope. This dissimilarity is not due to defects in any step of viral replication and was fully dependent on a competent immune system, since no differences in viral loads were observed in 5-Gy-x-ray-irradiated mice infected with either wild type F-MLV or the DM virus.

The authors unraveled the effectors responsible for the control of replication of the DM virus by using mice depleted of NK cells and athymic Swiss-Nude mice. In these experiments, they showed the involvement of NK1.1+ and CD8 T cells as respectively part of the innate and adaptive mechanisms in the early and late control of viral replication. Thus, both the innate and adaptive immunity are modulated by the IS domain.

The authors suggest that the DM virus could be considered a *bona fide* attenuated virus and could be useful for the design of new vaccine platforms. Swiss mice infected with the DM mutants do not develop viremia after challenge with wild type F-MLV. Interestingly, the antiviral responses are higher for the DM envelope than the F-MLV Env when C57BL/6 mice are injected with UV-inactivated viruses or when Swiss mice are injected with a 64 amino acid recombinant protein corresponding to the Env’s ectodomain that includes the IS region.

The study discussed above and previous work from the same investigators clearly show that in several retroviruses the “mechanical” fusogenic activity of Env protein that allows viral entry into the cell can be uncoupled from its immune-modulator function [1,9]. This is another example of how viruses with small genomes make use of smart strategies to survive in the adverse territory imposed by the host. The IS function of Env is conserved in phylogenetically distinct retroviruses, although not all retroviruses harbor this domain. Thus, retroviruses have developed alternative and redundant mechanisms that allow them to successfully establish infection.

In the future, it will be extremely important to understand precisely how the retroviral IS domain suppresses the host innate and adaptive immunity. The increased immunogenicity of inactivated DM mutants observed by the authors is a promising step forward for the improvement of antiretroviral vaccines and future studies will need to explore vigorously this lead.

## Figures and Tables

**Figure 1.**
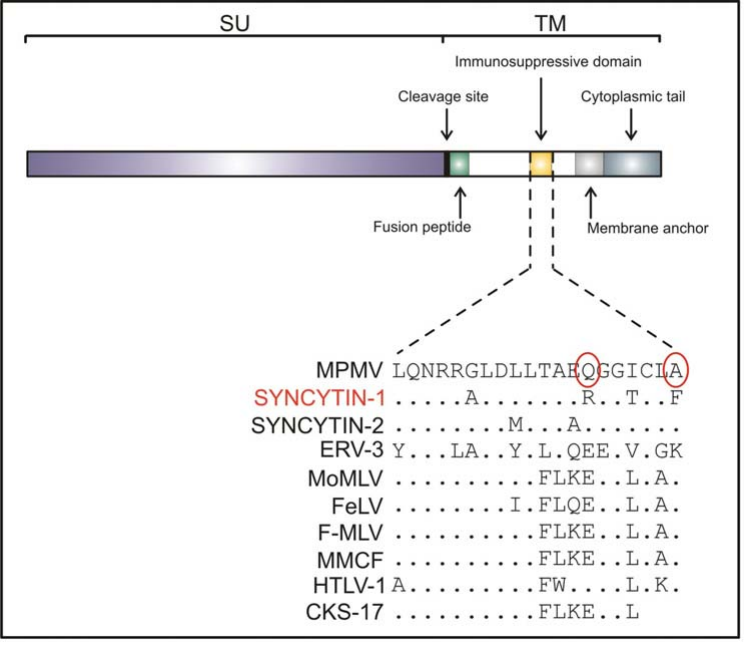
Domains of the retroviral envelope glycoprotein. Top: schematic representation of the domains of a retroviral envelope including the position of the putative immunosuppressive region (not drawn to scale). SYNCYTIN-1 is highlighted in red since it is not immunosuppressive. Bottom: Amino acid sequence alignment of the conserved immunosuppressive region of various retroviral envelope proteins. The amino acid substitutions of the double mutant are indicated with a red circle. MPMV, Mason Pfizer monkey virus (accession number: M12349); SYNCYTIN-1 (accession number: AY101585); SYNCYTIN-2 (accession number: NM_207582); ERV-3 (accession number: NM_001007253); MoMLV, Moloney murine leukemia virus (accession number: AF462057); FeLV, feline leukemia virus, (accession number: AY662447); F-MLV, Friend murine leukemia virus (accession number: M93134); MCF, mink-cell focus-forming virus (accession number: K02533); HTLV-1, human T cell lymphotropic virus (accession number: AY549882).
